# Wire Electrochemical Micromachining of Aluminum Rings for the Fabrication of Short-Millimeter Corrugated Horns

**DOI:** 10.3390/mi11020122

**Published:** 2020-01-22

**Authors:** Xiaolong Fang, Xiangyang Wang, Jiacheng Zhu, Yongbin Zeng, Ningsong Qu

**Affiliations:** National Key Laboratory of Science and Technology on Helicopter Transmission, Nanjing University of Aeronautics and Astronautics, Nanjing 210016, China; 15251765173@gmail.com (X.W.); nuaazjc@nuaa.edu.cn (J.Z.); binyz@nuaa.edu.cn (Y.Z.); nsqu@nuaa.edu.cn (N.Q.)

**Keywords:** electrochemical micromachining, aluminum, helical electrode, corrugated horn, electroforming

## Abstract

With the increase of working frequency, the feature size of a corrugated horn will be greatly reduced, causing challenges for fabrication. This paper investigated wire electrochemical micromachining (WECMM) of aluminum rings for assembly of a mandrel for electroforming, which has been a primary method for producing corrugated horns. By utilizing a rotary helical electrode and green additives, the removal efficiency of electrolytic products in WECMM was improved. It was found that the machined slits had good unilateral consistency on the left side of the electrode feeding direction when the electrode rotated clockwise. Complexing agent glutamic diacetic acid (GLDA) can compete with OH^−^ for Al^3+^ and has an obvious effect in reducing insoluble electrolytic products. From experimental investigations on typical parameters, an optimal parameter combination considering slit homogeneity and machining efficiency was obtained. In an electrolyte solution containing 15 g/L sodium nitrate solution and 15 g/L GLDA, 100 μm-thick aluminum rings with good edge and surface qualities were fabricated at a rate of 1.2 μm/s using a helical electrode with a diameter of 0.3 mm. Finally, these aluminum rings were successfully applied to make an internal corrugated sample with a rib width of 100 μm and a groove depth of 500 μm.

## 1. Introduction

Corrugated horns are widely utilized in various fields, for instance in space guidance, microwave remote sensing, satellite communication, radio astronomy, and target detection, owing to their outstanding advantages, including having low side lobes and low cross-polarization [1]. Mechanical milling, assembly, electroforming, and additive manufacturing are the typical methods for producing corrugated horns [2,3,4,5]. Recently, corrugated horns in the millimeter waveband have been primarily made of copper through electroforming, because this method has great advantages in terms of realizing internal microstructures and has the ability to transition from internal fabrication to surface fabrication using a mandrel [3,6].

An electroforming mandrel is usually made from an aluminum rod as a whole or is assembled from a set of aluminum rings. With the increase of working frequency in short-millimeter-band networks, the feature sizes of corrugated grooves and teeth in a horn and the corresponding mandrel will be critically decreased to micron dimensions, which would raise challenges for fabrication [7]. Many efforts have been made to produce microparts with corrugated microstructures on the horn’s cylindrical surface. Adams et al. [8] applied focused ion beams to generate microgrooves on cylindrical samples made of 6061-T6 aluminum, for which the feature dimensions were small enough but the surface had obvious burrs, which are not allowed in the electroforming process. Wang et al. [9] discussed the fabrication of a rotating microstructure using reciprocated wire electrical discharge micromachining, but the large corner radius at the bottom of the groove limited its application. In this way, the probable solution is to fabricate aluminum rings with micron thickness with no deformations or burrs.

Wire electrochemical micromachining (WECMM) removes materials at the atomic level, with no contact between the tool electrode and workpiece. Therefore, it has certain inherent characteristics, such as causing no residual stress, no lateral damage, and no metallurgical defects [10,11]. WECMM can machine metals regardless of its mechanical properties. All of these features make WECMM a reliable and flexible micromachining method that has drawn great attention. Employing a metallic wire as the cathode, WECMM has been used to successfully fabricate various microstructures, for example using grooves, gears, and helix coils [12,13,14]. However, the ability of WECMM to obtain process stability, acceptable surface quality, and acceptable machining efficiency is limited or diminished owing to insufficient fresh electrolyte renewal and product removal in a narrow machining gap [14,15]. Various methods have been proposed to enhance the electrolyte and product transfer process. By applying electrolyte flushing, Zeng et al. [16] successfully fabricated microstructures with feature heights up to 10 mm and feature aspect ratios of 50 or more. Xu et al. [17] and Meng et al. [18] utilized tool vibration and workpiece vibration with WECMM to accelerate electrolyte renewal, fabricated arrayed microtools, and high precision microstructures in metallic glass respectively.

In electrochemical micromachining, acids such as hydrochloric acid [17], sulfuric acid [18], and citric acid [19] were used as electrolytes to dissolve the hydroxide, although they are toxic and corrosive to aluminum. Another way to remove insoluble electrolytic products from the machining gap is to dissolve them through chemical reactions by adding complexing agents, as they have the ability to generate soluble products with metal ions [20,21]. Milosev et al. [22] revealed a pronounced effect on the passivation characteristics of stainless steel in physiological solution by adding EDTA (Ethylene Diamine Tetraacetic Acid) and citrate. Chen et al. [20] successfully fabricated microholes on SS304 plates with a depth of 400 μm in electrolyte-containing sodium chlorate and ethylene diamine tetra acetic acid disodium (EDTANa_2_). However, EDTA is hazardous to the operator due to it being strongly pungent.

In this paper, we utilized a rotary helical electrode during the WECMM process in order to improve the electrolytic product removal efficiency, whereby glutamic diacetic acid (GLDA) was added to the sodium nitrate electrolyte. GLDA is an environmentally friendly complexing agent and is produced using a green process involving the fermentation of readily available corn sugars. With the investigation of some key parameters in WECMM, micro-aluminum rings with a thickness of 100 μm were fabricated with no burrs or deformation. Finally, these microrings were applied to electroform a corrugated horn sample.

## 2. Methods

### 2.1. WECMM Aluminum Ring Utilizing A Rotary Helical Electrode

**Figure 1:**
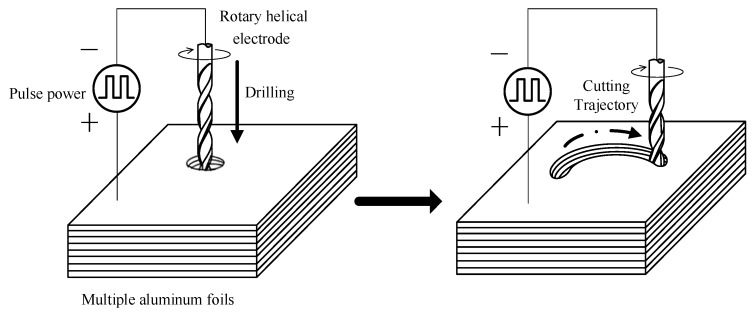
Schematic diagram of electrochemical micromachining of aluminum rings.

Rather using than a typical metallic wire, a rotary helical tool with spiral grooves on the surface was employed as the cathode for electrochemical micromachining in this paper. The effect of the rotary helical electrode was evaluated in our previous work [23]. The helical electrode attached to a spindle is fed along the programmed tool path to fabricate the expected structure. Figure 1 shows a schematic diagram of WECMM utilizing a rotary helical electrode to fabricate aluminum rings for the assembled mandrel in electroforming a corrugated horn antenna. First, the rotary electrode was utilized to drill a hole through the aluminum layers to create a starting point for follow-up trajectory cutting. Afterwards, the electrode was programmed to proceed along a circular path. These two steps are both electrochemical micromachining processes. Furthermore, anodic workpieces used in the machining were stacked together with several aluminum layers, among which the first layer takes the role of a sacrificial layer to protect other layers from corrosion. As a result, several aluminum rings could be machined at the same time, thus the machine efficiency was improved. During machining, the whole machining zone should be immersed by the electrolytes.

### 2.2. Effect of Complexing Agents in Electrochemical Reactions

Anodic reactions in electrochemical machining of aluminum with sodium nitrate solution are the dissolution of aluminum and reduction of dissolved oxygen. Thus, insoluble aluminum hydroxide is generated, which could be observed as white flocs in electrolyte during the machining process. They are the major electrolytic products needed to be removed from the machining zone.

(1) $$\mathrm{Al}-3e^{-}↔{\mathrm{Al}}^{3+},$$

(2) $$O_{2}+2H_{2}O+4e^{-}↔4{\mathrm{OH}}^{-},$$

**Figure 2:**
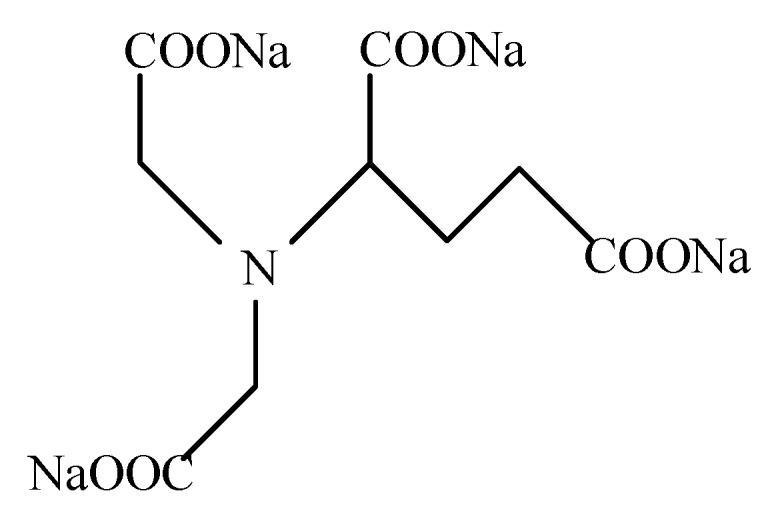
Chemical structural formula of glutamate diacetic acid (GLDA).

However, a different situation occurred when complexing agents were added to the electrolyte. The white flocs were no longer observed in the solution. The complexing agents utilized in this work were tetra sodium glutamate diacetic acid (GLDA), the ligand structure (L) of which is described in Figure 2. Negatively charged ligand L competes with OH^−^ for Al^3+^, thus aluminum hydroxide could be dissolved gradually due to the complexation effect:

(3) $$\mathrm{Al}{\left(\mathrm{OH}\right)}_{3}+3L^{-}↔{\mathrm{AlL}}_{3}+3{\mathrm{OH}}^{-},$$

According to the literature [21], there a competition coefficient can be used to evaluate to the generation of soluble clathrate:

(4) $$K_{j}=\frac{\left[AlL_{a}\right]\times {\left[OH^{-}\right]}^{n}}{{\left[L\right]}^{a}}=K_{sp}\left(Al{\left(OH\right)}_{n}\right)\times K_{f}\left(AlL_{a}\right),$$

where $K_{sp}\left(Al{\left(OH\right)}_{n}\right)$ presents the product solubility in water. Here, $K_{f}\left(AlL_{a}\right)$ is the stability constant logarithm of the clathrate $AlL_{a}$. The stronger the complexing ability of complexing agents, the more stable the generated clathrate under the same condition. Therefore, hydroxide precipitate would be dissolved smoothly if the complexing agents are strong enough.

## 3. Experimental

**Figure 3:**
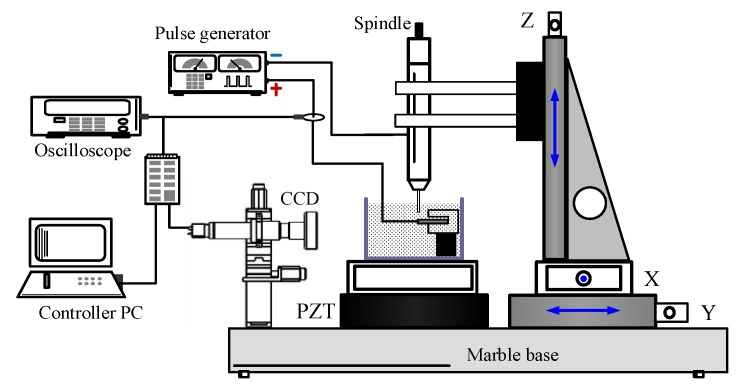
Schematic of experimental apparatus. Note: CCD = charge-coupled device; PZT = Piezoelectric Transducer.

A WECMM experimental system was established to conduct the experiments, as shown in Figure 3. An X-Y-Z-C motion stage, a PZT (Piezoelectric Transducer) motion stage, a pulse generator, an electrolyte unit, and an oscilloscope were contained in this system. The rotary helical electrode was mounted to the spindle, which moved along the programmed trajectory by X-Y-Z linear axes. The workpiece was made of ten stacked aluminum foil layers and was electrically connected to the negative pole of the power supply. In addition, the whole machining zone was observed by a charge-coupled device (CCD).

Specimens made of aluminum measured 15 mm × 10 mm × 0.1 mm and were cleaned ultrasonically before and after the experiments in ethanol solution. Pre-experiments were carried out to provide suggestions for good machining stability. Though temperature has a positive effect on electrolyte conductivity and influences material removal rate, the machining accuracy will decrease with the increase of electrolyte conductivity according to electrochemical machining theory. A low-conductivity electrolyte solution is usually applied in microfabrication. Here, the electrolyte concentration was chosen as 15 g/L and its temperature was kept at room temperature (maintained at 25 °C). Pulse frequency is an important parameter in electrochemical micromachining and has been well investigated in literature. A pulse frequency of 10 kHz was chosen from trial experiments considering the machining efficiency. Afterwards, experiments were conducted to investigate the effects of complexing agents, applied voltage, and electrode diameter on WECMM. Process conditions are listed in Table 1. When the machining reached an equilibrium status, the machining gap as well as the slit width remained constant. As the electrode feed rate was quite slow, each slit cutting experiment was stopped when the slit width reached a stable value for measurement. The slit lengths in the following figures are different. Dimensions of the machined slits and aluminum rings were measured via pictures captured by a scanning electron microscope (SEM) (S-3400N, Hitachi, Tokyo, Japan).

## 4. Results and Discussion

### 4.1. Surface Difference of Two Sides of Machined Slits

**Figure 4:**
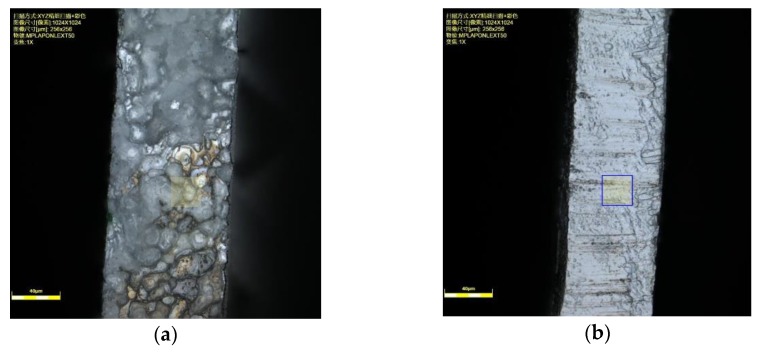
Photographs of two sides of the machined slit: (**a**) rough side of the slit, Ra 1.427 μm; (**b**) fine side of the slit, Ra 0.391 μm.

The slit width and its homogeneity are usually taken as an important index for evaluating the machining accuracy of WECMM. The pre-experiments indicated that the machined slit had the characteristic of good unilateral consistency; namely, two sides of the machined slits were different. To measure the surface difference, the machined slit was cut along the middle line and the two parts were observed by laser-scanning confocal microscopy. Figure 4 shows the photographs of two sides of the machined slit. The surface roughness is Ra 1.427 μm on the rough side, while it is Ra 0.391 μm on the fine side.

**Figure 5:**
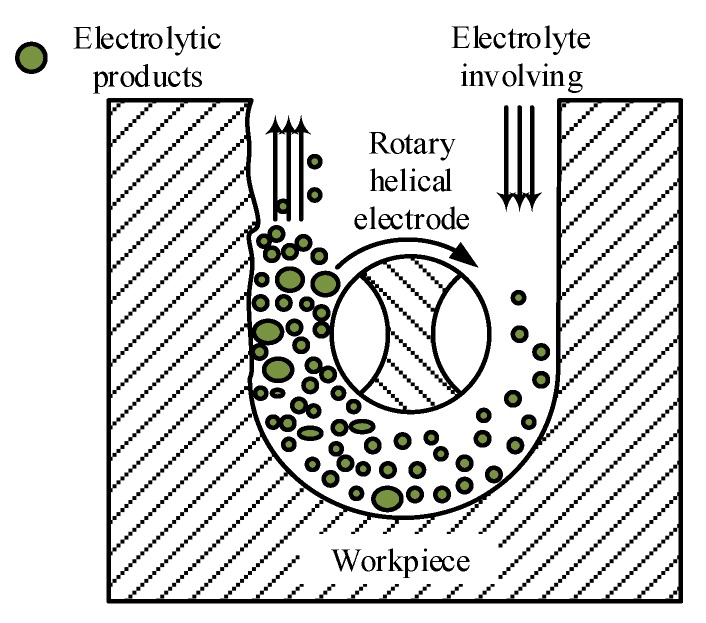
The transfer mechanism of the electrolytic products in the machining gap.

The electrolyte transfer mechanism in the machining gap with the rotary helical electrode is illustrated in Figure 5. As the rotary helical electrode keeps rotating in the machining gap, fresh electrolyte solution is involved in the machining zone and evenly contacts the workpiece, leading to a homogeneous slit width on this side. However, as the electrochemical reaction proceeds, dirty electrolyte solution containing massive electrolytic products is extruded out from the frontal machining gap to the other side. The electrolyte conductivity on this side is significantly affected by the electrolysis products. The relationship between electrical conductivity *κ* and the gas void fraction *β*_gas_ is given by:

(5) $$\kappa =\kappa_{0}{\left(1-\beta_{gas}\right)}^{bp}(1+\alpha (T-T_{0}))$$

where *bp* represents Bruggeman’s coefficient. When electrolytic products are extruded out, massive bubbles are produced on this side as well, resulting in the increase of the gas void fraction, $\beta_{gas}$. Thereby, the electrolyte conductivity in this machining zone decreases. The inhomogeneous distribution of electrolyte conductivity in the machining gap will cause uneven material removal and poor surface integrity on this side. A similar phenomenon has also been found in machining nickel alloys [24], but the phenomenon is extremely serious in machining aluminum as it is sensitive to electrolytes.

**Figure 6:**
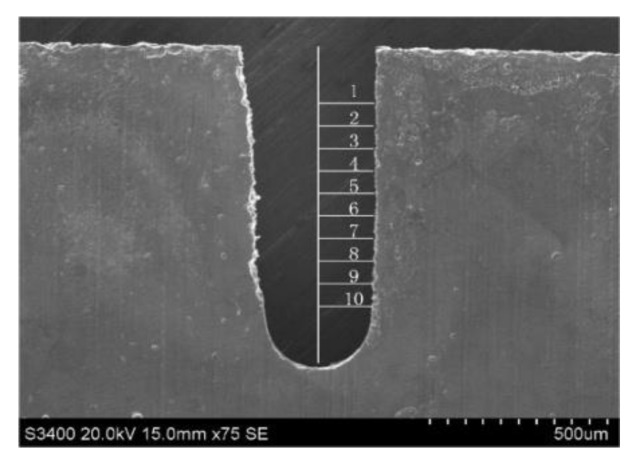
The measurement of unilateral slit width.

As only one side of the machined slit forms the inner or outer edge of an aluminum ring, we chose the side with good surface integrity to evaluate the process performance. That is, measurements of slit width were carried out according to the distance form this better surface to the centerline of the slit. Ten measurements at different positions produced an average value for the unilateral slit width, as shown in Figure 6. Each standard deviation of slit width was calculated according to these ten values, revealing the slit width consistency.

### 4.2. Effect of Applied Voltage

**Figure 7:**
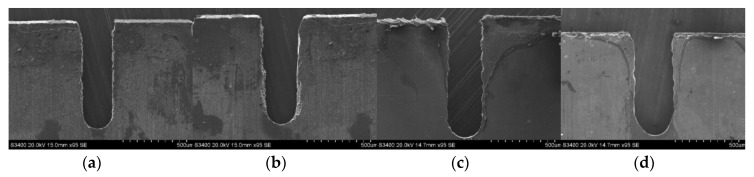
The slits on aluminum layers machined by different applied voltages: (**a**) 7.0 V; (**b**) 7.5 V; (**c**) 8.0 V; (**d**) 8.5 V.

**Figure 8:**
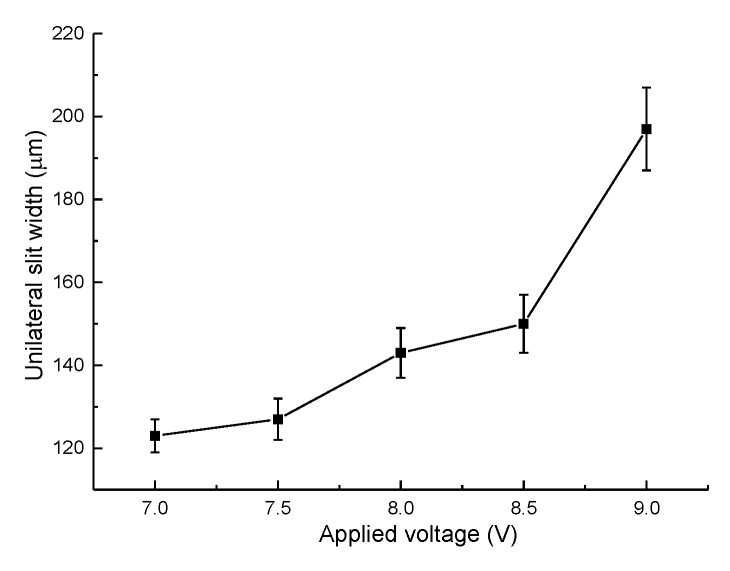
Variation of the unilateral slit width with applied voltage.

The slits on aluminum layers machined by different voltages applied between the helical electrode and workpiece are presented in Figure 7. Figure 8 presents the variation of the unilateral slit width with applied voltage. The electrode is of 0.2 μm in diameter, the electrode feed rate is 0.2 μm/s, and the solution is 15 g/L sodium nitrate added to 15 g/L of GLDA.

As shown in Figure 8, the unilateral slit width increased dramatically with the increase of applied voltage. According to Faraday’s law and Ohm’s laws, as the applied voltage increases, the current density applied in the machining gap increases accordingly, resulting in a larger material removal rate (MRR). Therefore, a wide slit could be observed in this experiment with an applied voltage of 9 V. Additionally, stray corrosion is serious under a large voltage, causing unwanted material removal and terrible machining surfaces. However, when the applied voltage was lower than 7 V, frequent short circuits were observed. In electrochemical machining, the equilibrium machining gap is an essential index and can be calculated by:

(6) $$\Delta_{b}=\eta \omega \kappa \frac{U-\delta_{e}}{v_{f}},$$

where *η* is the current efficiency of anodic dissolution, *ω* is the volumetric electrochemical equivalent, *κ* is the electrolyte electrical conductivity, *U* is the applied voltage, *δ*_e_ is polarization potential, and *v_f_* is the electrode feed rate. Therefore, a low applied voltage could lead to a narrow machining gap when other conditions are fixed. With the decrease of machining gap, the removal of massive electrolytic products would be more difficult. Insufficient product removal would cause uneven distribution of electrical conductivity and deteriorate the continuous material dissolution. As a result, 7 V was recommended as the optimized applied voltage in this context.

### 4.3. Effect of Electrode Diameter

Here, we used the slit homogeneity and the machining efficiency as the evaluation indexes. The electrode diameter is probably the most significant factor relating to the slit width and its homogeneity. Two electrodes with a different diameter of 0.2 mm and 0.3 mm were experimented on to verify their effects on the slit width homogeneity and machining efficiency, respectively. The solution contained 15 g/L of sodium nitrate added to 15 g/L of GLDA.

**Figure 9:**
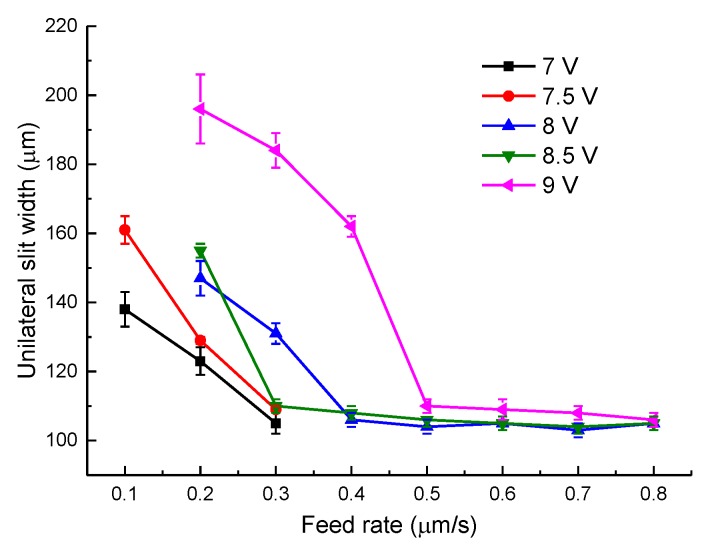
The variation of unilateral slit width with electrode feed rate, utilizing an electrode measuring 0.2 mm in diameter.

**Figure 10:**
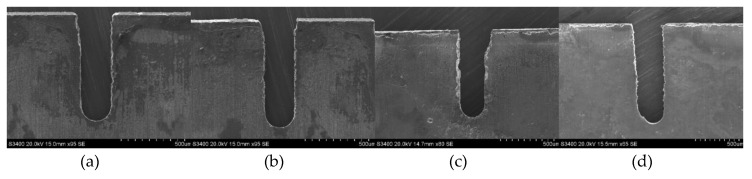
The slits machined by voltage feed rate combinations, utilizing an electrode diameter of 0.2 mm: (**a**) 7 V–0.3 μm/s; (**b**) 7.5 V–0.3 μm/s; (**c**) 8 V–0.7 μm/s; (**d**) 8.5 V–0.8 μm/s.

Figure 9 shows the variation of the unilateral slit width with electrode feed rate, utilizing an electrode with a diameter of 0.2 mm under different applied voltages. This indicated that with the increase of feed rate, the machined slit width was narrower under different applied voltages. The change trend matched the equation for the machining gap in WECMM, which is given as:

(7) $$\Delta_{s}=\sqrt{2D\Delta_{b}+\Delta_{b}^{2}},$$

where *D* is the diameter of the electrode and Δ_b_ is the equilibrium machining gap. As the feed rate increased, the machining balance gap Δ_b_ decreased according to Equation (6), therefore the machining gap Δ_s_ also decreased according to Equation (7). However, when the feed rate remained increasing, the machining gap Δ_b_ became too narrow for the removal of electrolytic products, causing frequent short circuits, which has been discussed above. Hence, different applied voltages had different maximum feed rates, the combinations of which are listed as: 7 V–0.3 μm/s, 7.5 V–0.3 μm/s, 8 V–0.7 μm/s, 8.5 V–0.8 μm/s, and 9 V–0.8 μm/s. Figure 10 presents the slits machined with some of the above combinations, among which 7.5 V–0.3 μm/s showed the best slit width homogeneity. However, the machining efficiency of this combination was too low to meet our expectations.

**Figure 11:**
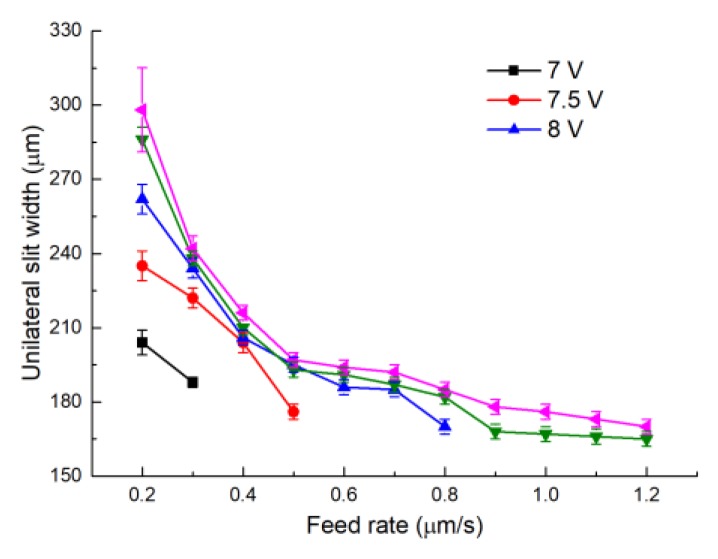
The variation of unilateral slit width with electrode feed rate, utilizing an electrode measuring 0.3 mm in diameter.

**Figure 12:**
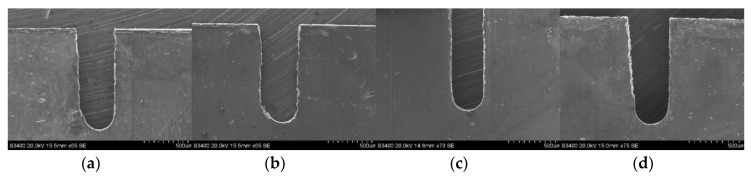
The slits machined by voltage feed rate combinations utilizing an electrode diameter of 0.2 mm: (**a**) 7 V–0.3 μm/s; (**b**) 7.5 V–0.5 μm/s; (**c**) 8.5 V–1.2 μm/s; (**d**) 9 V–1.2 μm/s.

Figure 11 presents the variation of the unilateral slit width with feed rate, utilizing an electrode with a diameter of 0.3 mm under different applied voltages. The conditions were the same as with an electrode diameter of 0.2 mm. Generally, the plots show the same trend as with the electrode with a diameter of 0.2 mm. However, as the electrode diameter increased, the top feed rate also increased. The reason is that a larger helical electrode has a higher linear velocity with which to drag the electrolyte solution into the machining gap. Thereafter, the efficiency of electrolyte refreshment and product removal can be significantly enhanced. This allows the machining process to proceed in a stable manner with a higher material removal rate, as well as a high feed rate. Hence, the maximum electrode feed rate increased under a larger electrode diameter. The combinations of top feed rates under different applied voltages are 7 V–0.3 μm/s, 7.5 V–0.5 μm/s, 8 V–0.9 μm/s, 8.5 V–1.2 μm/s, 9 V–1.2 μm/s. Figure 12 presents slits machined under some of these combinations. It is obvious that the 8.5 V–1.2 μm/s combination had the most uniform slit. Additionally, this combination had the advantage of a high feed rate, which met the requirements for machining efficiency.

### 4.4. Effect of GLDA Concentration

**Figure 13:**
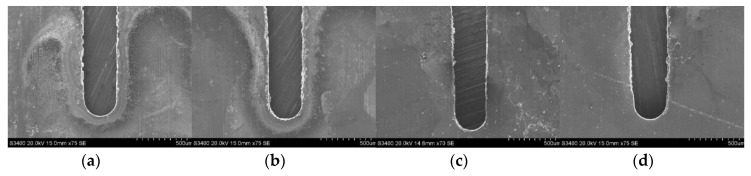
The slits machined by different GLDA concentrations: (**a**) 0 g/L; (**b**) 7.5 g/L; (**c**) 15 g/L; (**d**) 30 g/L.

**Figure 14:**
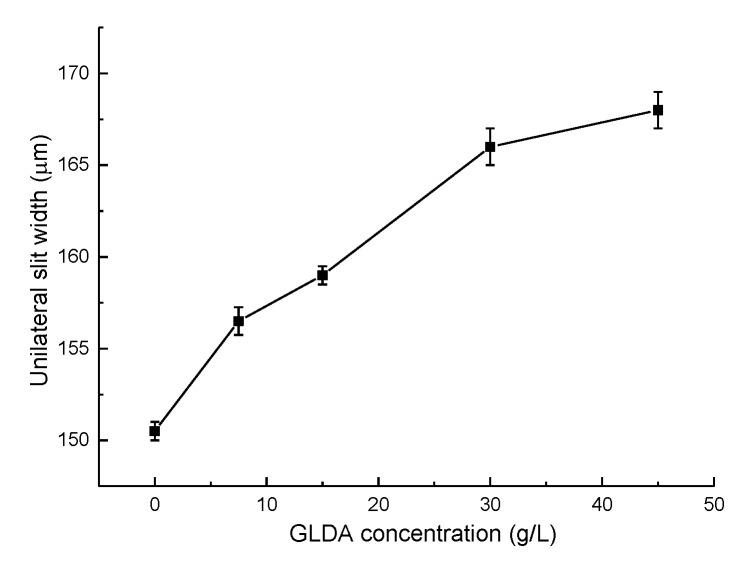
The variation of the unilateral slit width with GLDA concentration.

As mentioned in Section 2.2, the addition of GLDA could have positive effects on avoiding the appearance of insoluble electrolytic products. The machined slits manufactured with different GLDA concentrations are presented in Figure 13. The machining parameters are 8.5 V in applied voltage, 0.3 mm in electrode diameter, and 1.2 μm/s in electrode feed rate. The change of unilateral slit width with GLDA concentration is illustrated in Figure 14. Massive white flocs were observed when there was no addition of GLDA, and the electrolyte solution became feculent quickly. The oscilloscope detected frequent short circuit signals due to the blocking of electrolytic products in the machining gap. Therefore, the slit homogeneity was extremely poor, and the workpiece surface was covered in white film as well. However, a different situation occurred with the addition of GLDA in the electrolyte solution. The ligand structure in the GLDA competed with OH^−^ for Al^3+^, and rare insoluble products were observed. The more GLDA present, the stronger the ligand structure according to Equation (4). In addition, the homogeneity of the slit width was better with even current distribution in the machining gap due to the absence of dirty electrolytic products. Unfortunately, the quantity of conducting ions in the electrolyte increased with the GLDA concentration. Therefore, the electrolyte conductivity as well as the current density in the machining gap increased, causing a wider slit. The addition of 15 g/L GLDA to the sodium nitrate solution was recommended according to its smallest width deviation.

### 4.5. Fabrication of Aluminum Ring

**Figure 15:**
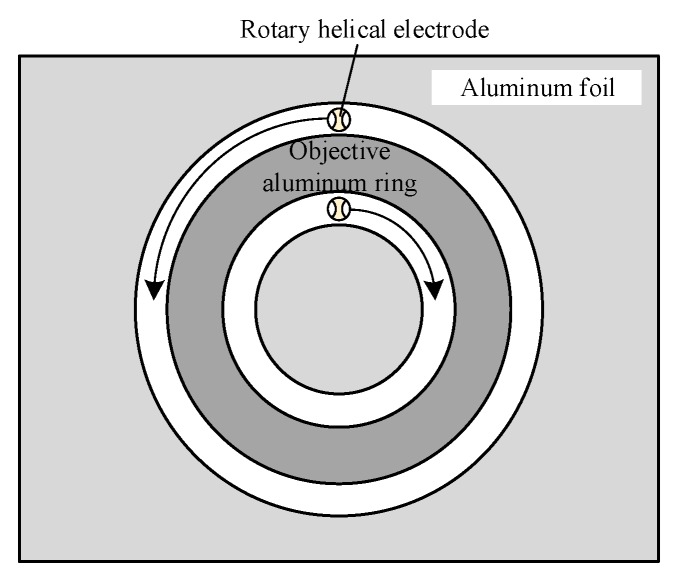
The feed trajectories of the outer circle and inner circle.

The fabrication of aluminum rings consists of inner edge machining and outer edge machining. As mentioned above, the slits machined by a rotary helical electrode had good unilateral consistency, with better side results occurring to the left of the electrode feed direction. In order to obtain aluminum rings of high quality, the outer edge and inner edge should be machined in line with the better slit sides. Figure 15 illustrates the feed trajectories of outer and inner machining edges. It is obvious that the inner edge should be machined in a clockwise direction, while the outer should be machined in an anti-clockwise direction.

**Figure 16:**
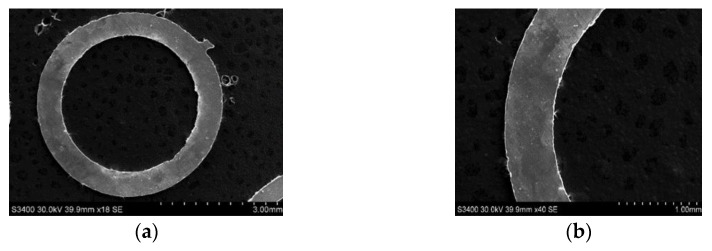
The scanning electron microscopy (SEM) photographs of the final aluminum ring: (**a**) integral photograph; (**b**) local photograph.

According to the experimental investigations above, 100 μm-thick aluminum rings with good edge and surface qualities were successfully machined using a helical electrode with a diameter of 0.3 mm, as shown in Figure 16. The electrolyte was the 15 g/L sodium nitrate solution with 15 g/L GLDA; the pulsed voltage was 8.5 V in amplitude, 10 kHz in frequency, with a 10% duty ratio; the spindle speed was 2000 rpm; and the electrode feed rate was 1.2 μm/s.

## 5. Electroformed Corrugated Horn with An Assembled Mandrel

**Figure 17:**
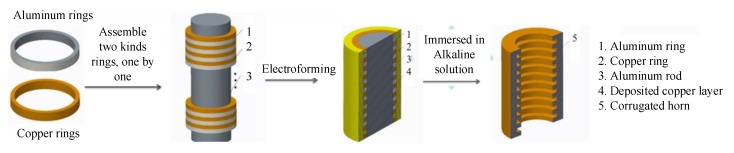
Schematic diagram of electroforming a corrugated horn.

**Figure 18:**
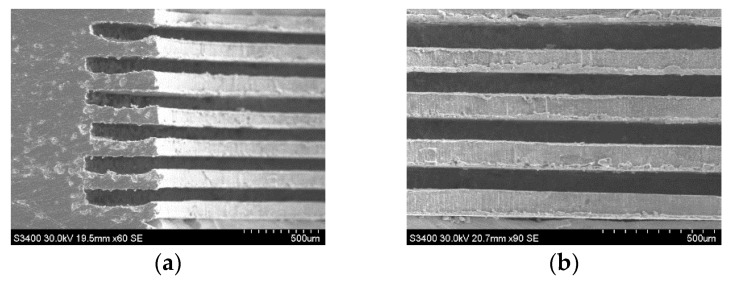
The SEM photographs of internal corrugated structures: (**a**) cross-section view; (**b**) enlarged view.

As mentioned in Section 1, electroforming is a primary method used to fabricate corrugated horns. Figure 17 illustrates the schematic diagram of electroforming with a mandrel assembled from copper and aluminum rings. Under the conditions listed in Table 2, a sample with internal corrugated structures was fabricated, as shown in Figure 18. It had a rib width of 100 μm and a groove depth of 500 μm. This verified the feasibility of wire electrochemical machining of aluminum rings for the fabrication of corrugated horns. More efforts should be made to improve the machining efficiency for industrial production.

## 6. Conclusions

A WECMM method utilizing a rotary helical electrode has been proposed to fabricate aluminum rings. The effects of complexing agents and typical parameters on slit size and surface quality were investigated. The conclusions can be summarized as follows:

(1) Slits machined by a rotary helical electrode had good unilateral consistency on the left side of the electrode feeding direction when the electrode rotated clockwise. 

(2) Complexing agent GLDA competed with OH^−^ for Al^3+^ during electrochemical machining of aluminum and had an obvious role in reducing insoluble electrolytic products.

(3) An optimal parameter combination considering slit homogeneity and machining efficiency was obtained. In the 15 g/L sodium nitrate solution with 15 g/L GLDA added, a helical electrode with a diameter of 0.3 mm and a rotation speed of 2000 rpm was fed at 1.2 μm/s. The 100 μm-thick aluminum rings with good surface quality were successfully machined at 8.5 V. 

(4) A sample of internal corrugated structures with a rib width of 100 μm and a groove depth of 500 μm was electroformed using the mandrel assembled from the aforementioned aluminum rings.

## Figures and Tables

**Table 1:** Experimental conditions for wire electrochemical micromachining (WECMM)**.**

Parameters	Value
Electrolyte	15 g/L NaNO_3_
Glutamate diacetic acid (GLDA) (g/L)	0, 7.5, 15, 30, 45
Applied voltage (V)	7, 7.5, 8, 8.5, 9
Electrode diameter (mm)	0.2, 0.3
Duty ratio (%)	10
Electrode rotation speed (rpm)	2000
Electrode feed rate (μm/s)	0.1~1.2

**Table 2:** Conditions for electroforming.

Parameter	Value
CuSO_4_ (g/L)	80
H_2_SO_4_ (g/L)	150
Temperature (°C)	45
Current density (A/dm^2^)	2
Mandrel rotation speed (rpm)	30
